# Understanding allostasis: Early‐life self‐regulation involves both up‐ and down‐regulation of arousal

**DOI:** 10.1111/cdev.14136

**Published:** 2024-07-26

**Authors:** S. V. Wass, F. U. Mirza, C. Smith

**Affiliations:** ^1^ Department of Psychology University of East London London UK; ^2^ Institute of Psychiatry, Psychology & Neuroscience King's College London London UK

## Abstract

Optimal performance lies at intermediate autonomic arousal, but no previous research has examined whether the emergence of endogenous control associates with changes in children's up‐regulation from hypo‐arousal, as well as down‐regulation from hyper‐arousal. We used wearables to take day‐long recordings from *N* = 58, 12‐month‐olds (60% white/58% female); and, in the same infants, we measured self‐regulation in the lab with a still‐face paradigm. Overall, our findings suggest that infants who showed more self‐regulatory behaviors in the lab were more likely to actively change their behaviors in home settings moment‐by‐moment “on the fly” following changes in autonomic arousal, and that these changes result in up‐ as well as down‐regulation. Implications for the role of atypical self‐regulation in later psychopathology are discussed.

**Abbreviations**
ANS: autonomic nervous system
ECG: electrocardiogram
PACF: partial autocorrelation function
RMSSD: root mean square of successive differences

Freud first defined the central nervous system as: “an apparatus which has the function of getting rid of the stimuli that reach it, or of reducing then to the lowest possible level; or which, if it were feasible, would maintain itself in an altogether unstimulated condition” (Freud & Strachey, 1900). Since then, many researchers who examine the development of regulation have, implicitly, followed Freud's approach. Hypo‐arousal as a trait‐level feature has been discussed in the context of developmental psychopathologies such as attention deficit hyperactivity disorder (Bellato et al., 2020) and psychopathy (Verona et al., 2004). But researchers who study active regulatory processes, defined as “the ongoing, dynamic, and adaptive modulation of internal state (emotion, cognition) or behaviour, mediated by central and peripheral physiology” (Nigg, 2017), mainly tend to study how children down‐regulate arousal in response to hyperstimulation of the autonomic nervous system (ANS), through behaviors such as gaze aversion, physical self‐soothing, and distraction (Kopp, 1982; Posner & Rothbart, 2000). For example, most widely used questionnaire measures of self‐regulation assess how quickly a child soothes following crying or distress (Derryberry & Rothbart, 1988); and lab‐based assessments measure a child's behavioral and physiological responses to a stressor such as arm restraint, or the still‐face procedure (Gagne et al., 2011)—both of which are responses to hyper‐arousal. But neither questionnaires nor laboratory assessments typically measure how children change their behaviors in response to hypo‐arousal.

There are, of course, a number of reasons why this is the case. Focusing on behavioral down‐regulation has been fruitful, as relevant questionnaire measures based on this approach reliably predict later‐life psychopathology and cognitive outcomes (Eisenberg et al., 2019; Kostyrka‐Allchorne et al., 2020; Nigg, 2017). But this concentration on behavioral responses to hyper‐arousal is nevertheless striking given that animal and human research suggests that, in fact, optimal cognitive performance lies at intermediate level of ANS activity, with both hypo‐ (under) as well as hyper‐ (over) arousal associated with poorer cognitive performance (Aston‐Jones & Cohen, 2005; Porges, 2007; Porges & Furman, 2011; Wass, 2021; Yerkes & Dodson, 1908). Historically, theorists have suggested, consistent with this, that there is an optimal level of stimulation toward which organisms strive (Berlyne, 1960; Dunn, 2002; Fiske & Maddi, 1961; Piccardi & Gliga, 2022; Porges, 2007; Schneirla, 1959; Wachs, 1977; Zentall & Zentall, 1983; Zuckerman, 1979), with any deviation from optimal stimulation (hypo as well as hyper) leading to aversive states for which individuals need to compensate via allostasis (the active process through which homeostasis is maintained by an organism, Cannon, 1929; McEwen & Wingfield, 2003). For example, Gardner, Karmel and colleagues measured how young infants' preference for less‐arousing, low‐frequency visual stimuli versus more‐arousing, high‐frequency visual stimuli varied contingent on their own arousal (Gardner & Karmel, 1984, 1995). They found that highly aroused 1‐month‐old infants preferred less arousing, low‐frequency stimuli, whereas less‐aroused infants preferred more‐arousing, high‐frequency stimuli (Gardner & Karmel, 1984, 1995). These results suggest that typically developing young infants dynamically recalibrate their attentional behaviors to up‐regulate their own arousal when it is low, as well as to down‐regulate their own arousal when it is high (see also Cole, Ram, et al., 2019; Cole, Ramsook, et al., 2019; Morales et al., 2018). Other research has discussed similar allostatic mechanisms in the context of caregiver–child dyads (Lunkenheimer et al., 2020; Somers, Curci, et al., 2021; Somers, Luecken, et al., 2021; Wass et al., 2019).

An approach that studies up‐ as well as down‐regulation of arousal will allow us to study how optimal levels of autonomic arousal may differ between individuals, a possibility that has been suggested (Berlyne, 1960; Fiske & Maddi, 1961; Wass, 2021; Zuckerman, 1979) but not, to our knowledge, studied (although see, e.g., Somers, Luecken, et al., 2021). If true, this would have fundamental consequences for how we understand self‐regulation. For example, if a child with adverse life experiences is assessed via questionnaire or laboratory task as not soothing quickly following an external stimulus which induces hyper‐arousal, this may be because they are trying to down‐regulate and failing; or, it may be because they are using allostasis effectively, but to maintain the higher level of ANS arousal that is, for them, optimal (see also Bellato et al., 2020).

Previous observational and experimental research has suggested even neonates can show some down‐regulatory behaviors (Brazelton, 1983; Stifter & Braungart, 1995). But little previous research has examined whether, and if so how, the capacity to actually use allostatic self‐regulation in real‐world settings develops across early development (Cole et al., 2020; Cole, Ramsook, et al., 2019; Wass, 2021). Here, we examine this, concentrating in particular on examining up‐ as well as down‐regulatory patterns at 12 months, which is thought to be the age when endogenous control can be robustly measured for the first time (Colombo & Cheatham, 2006; Wass, 2021). Furthermore, we examine how the prevalence of real‐world allostatic processes relates to self‐regulation as measured traditionally assessed in the lab. To do this, we first obtained day‐long naturalistic recordings using wearable sensors microphones, cameras, and physiological monitors (measuring heart rate, heart rate variability, and movement). Then, to the same infants, we administered a standard mild stressor (a still‐face procedure, Weinberg & Tronick, 1996) to a cohort of typical infants, and coded self‐regulation in the traditional manner, by video‐coding down‐regulatory behaviors such as closing eyes, gaze aversion, and physical self‐soothing (see “Methods” section for more details).

In comparing, for the first time, associations between lab‐assessed self‐regulation and self‐regulation and autonomic arousal fluctuations in real‐world settings, we had three main questions. First, is better self‐regulation (as assessed using a traditional approach in a lab setting) associated with lower arousal overall in real‐world settings, and changes in affect and intensity? Second, is better self‐regulation in lab settings associated with differences in the rate of change of affect, intensity, and arousal in real‐world settings (see Somers & Luecken, 2022; Somers, Luecken, et al., 2021)? Third, is better self‐regulation in lab settings associated with greater evidence for the existence of allostatic processes—and, specifically, increased up‐regulation from hypo‐arousal in real‐world settings?

One specific behavior that we examined in detail is spontaneous infant vocal behaviors. Previous research based on the same dataset has suggested that, consistent with theoretical predictions (Kopp, 1982), clusters of vocalizations (both cries and speech‐like vocalizations) tend overall to occur during periods of elevated infant arousal (Wass et al., 2019, 2022). Cries occur following reduced infant arousal stability and elicit changes in caregiver behavior (Wass et al., 2019), which lead to arousal down‐regulation via co‐regulation (Wass et al., 2022). Speech‐like vocalizations, which are neutral in affect, also occur at elevated arousal, but lead to longer‐lasting increases in arousal, and elicit more parental verbal responses (Wass et al., 2022).

In part 1 of our analyses, we examine our data overall. We predicted that increased lab‐assessed self‐regulation would associate with: reduced overall arousal in naturalistic settings; reduced vocal affect and intensity in real‐world settings; a slower rate of change of arousal and reduced variability in vocal affect and intensity in naturalistic settings; and faster recovery following “peak” arousal events.

In part 2 of our analyses, we examine how infants' responses and behaviors differ contingent on hypo‐ versus hyper‐arousal. First, we examined how the stability of arousal (i.e., the likelihood of being in the same state at time *t* + 1 as at time *t*) varies contingent on a child's arousal level at time *t*. Furthermore, we examined how these relationships differ contingent on lab‐assessed self‐regulation. We predicted that children with better self‐regulation (as assessed using conventional approaches in the lab) would show both more down‐regulation from hyper‐arousal and more up‐regulation from hypo‐arousal. Second, we tested how infants' responses to hyper‐ and hypo‐stimulation differ contingent on lab‐assessed self‐regulation by using dynamical generative models. Our predictions were the same as for the first hypothesis. Third, we examined how the relationship between vocalization likelihood and arousal, and the effects of vocalizing on arousal, differed contingent on lab‐assessed self‐regulation. We predicted that, in children with better lab‐assessed self‐regulation, their autonomic arousal would be more predictive of vocal behaviors.

## METHODS

### Participants

Participants were recruited by researchers visiting infant–parent groups, both privately organized and through local councils, as well as by mail by purchasing names and addresses of families in specific income brackets and areas of the South‐East area of the UK. Exclusion criteria were as follows: complex medical conditions, skin allergies, heart conditions, parents below 18 years of age, and parents receiving care from a mental health organization or professional. We also excluded families in which the primary day‐time care was performed by a male parent because the numbers were insufficient to provide an adequately gender‐matched sample. To assess demographics and maternal characteristics, we asked parents to self‐report occupation, education, and income. To assess maternal anxiety and depression, we administered the general anxiety disorder‐7 (GAD‐7) (Spitzer et al., 2006) and patient health questionnaire‐9 (PHQ‐9) (Kroenke et al., 2001) questionnaires. Data collection was conducted between 2018 and 2021.

Infants taking part in the study were recruited from the South‐East regions of the UK. Usable data from both components of the study—the lab visit and home visit—were obtained from 58 infants (mean (SE) age in days: 317.8 (4.4)). Demographic details for the sample, including the entire sample and the sample from whom usable data were obtained, are given in Table S1. A further *N* = 15 took part but failed to provide enough data to take part in the study (a full comparison of participants who did and did not provide usable data is included in Table S2).

### Equipment and procedures

#### Home visit

##### Protocol

The lab and home testing sessions were scheduled within median (SE) of 17.5 (3.5) days of one another. For the home visit, parents selected a day for which they would be spending the entire day with their child but that was otherwise, as far as possible, typical. The researcher visited the participants' homes in the morning (between 7:30 a.m. and 10:00 a.m.) to fit the equipment and explain its use, and then returned in the late afternoon (between 4:00 p.m. and 7:00 p.m.) to remove it. Mean (SD) recording time per day was 7.3 (1.4) hours. Participants reported no discomfort and disruption to their normal routines from wearing the equipment.

##### Equipment

**FIGURE 1:**
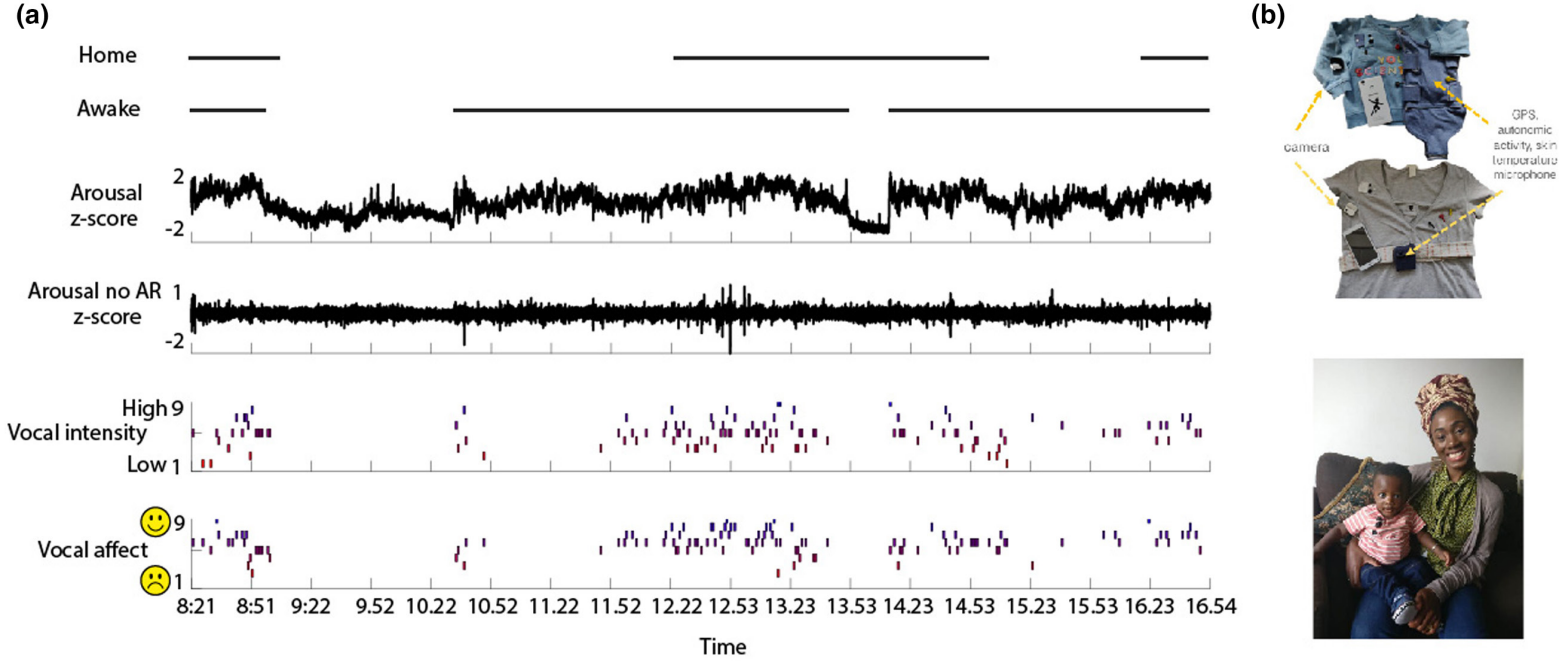
(a) A sample plot of raw data collected from a single participant showing (from top to bottom): the home/awake coding (conducted as described in the “Methods” section); the arousal composite score (calculated as described in the “Methods” section); the arousal composite score after removal of the autocorrelation (as described in the “Methods” section); the vocal intensity coding; and the vocal affect coding. (b) Photographs showing the equipment (top) and the same equipment being worn by some participants (bottom).

The equipment consisted of two wearable layers (see Figure 1b). A specially designed baby grow was worn next to the skin, containing a built‐in electrocardiogram (ECG) recording device (recording at 250 Hz), accelerometer (30 Hz), GPS (1 Hz), and microphone (11.6 kHz). A T‐shirt, worn on top of the device, contained a pocket to hold the microphone and a miniature video camera (a commercially available Narrative Clip 2). The clothes were comfortable when worn and, other than a request to keep the equipment dry, participants could behave exactly as they would on a normal day. No discomfort in wearing the equipment was generally reported. To ensure good‐quality recordings, the ECG was attached using standard Ag‐Cl electrodes placed in a modified lead II position.

##### Autonomic monitoring

Autonomic arousal was indexed by recording electrocardiography, from which heart rate and heart rate variability were derived, and actigraphy.

Data were parsed to identify the time intervals between the R peaks of the ECG signal using custom‐built Matlab scripts, employing an adaptation of a standard thresholding procedure (Wass et al., 2015), and verified post hoc via visual inspection. Further details of the parsing procedure are given in the Supporting Information (Section S1.2; Figures S1 and S2).

Heart rate variability was calculated using the PhysioNet Cardiovascular Signal Toolbox (Vest et al., 2018). A 60‐s window with an increment of 60 s was implemented, and the default settings were used with the exception that the min/max inter‐beat interval was set at 300/750 ms for the infant data and 300/1300 ms for the adult data. Heart rate variability was calculated from the root mean square of successive differences (RMSSD) (see further details in Section S1.3.1).

To parse the actigraphy data, the data were first manually inspected, then corrected for artifacts specific to the recording device used. Following that, a Butterworth low‐pass filter with a cut‐off of 0.1 Hz was used to remove high‐frequency noise (see further details in Section S1.3.2).

The preliminary analyses suggested that the three autonomic measures showed strong patterns of tonic and phasic covariation, consistent with previous research (Wass et al., 2015, 2016). See further details, and discussion, in the Supporting Information (Section S1.4; Figure S4). Motivated by this, and by analogous findings in the animal literature (Calderon et al., 2016; Pfaff, 2018), we collapsed the autonomic indices into a single composite measure.

Our analyses suggested that when infants were outside they were often strapped into a buggy or car seat, which strongly affected their autonomic data. Because of this, only segments of the data when infants were at home were included. Since all aspects of autonomic arousal differ markedly between waking and sleeping (see, e.g., Figure 1), sections where infants were asleep were also excluded. Details of the criteria through which home/not‐home and sleeping/waking segments were identified are given in the Supporting Information (Section S1.5). Following these exclusions, the mean (SD) amount of data entered into the analyses was 3.7 h (1.7 h) per participant.

##### Vocal affect and intensity coding

For reasons of bandwidth, the microphone recorded just a 5‐s snapshot of the auditory environment every 60 s. To assess the impact of this “sparse sampling” on our data, we performed a separate control analysis (see Section S1.6; Figure S4), which suggested that the temporal structure of our vocalizations was maintained despite this “sparse sampling” approach. Furthermore, since our analyses examine average patterns of arousal change around observed vocalizations, we reasoned that any observed changes would be weakened (not strengthened) by the fact that the vocalization data were sparsely sampled (because signal to noise could only be increased by missing vocalizations).

Post hoc, trained coders listened to each recording to identify samples in which the infant was vocalizing. The infant vocalizations were also coded for vocal affect on a scale from 1 (fussy and difficult) to 5 (happy and engaged), and for vocal intensity on a scale from 1 (mild) to 5 (intensely engaged). In order to assess inter‐rater reliability, 24% of the sample was double coded; Cohen's kappa for this coding was .60, which is acceptable (McHugh, 2012). All coders were blinded to all intended analyses. The relationship between vocal affect and intensity among all vocalizations is shown in Figure 3d.

##### Vocalization type coding

A morphological coding scheme (Oller et al., 2013) was applied with the following categories: cry, laugh, squeal, growl, quasi‐resonant vowel, fully resonant vowel, marginal syllable, and canonical syllable. Overall, 29% of vocalizations were cries; 1% laughs; 1% squeal; 3% growl; 18% quasi‐resonant vowel; 18% fully‐resonant vowel; 6% marginal syllable; and 23% canonical syllable. These were collapsed into cries and speech‐like vocalizations, which included the following non‐cry categories: quasi‐resonant vowel; fully resonant vowel; marginal syllable; and canonical syllable. Laughs, squeals, and growls were excluded due to rarity. In order to assess inter‐rater reliability, 11% of the sample was double coded, to assess the consistency of the cries (1) versus speech‐like vocalizations (0) distinction on which the analysis was based; Cohen's kappa was .70, which is considered substantial agreement (McHugh, 2012).

##### Permutation‐based temporal clustering analyses

To estimate the significance of time‐series relationships, a permutation‐based temporal clustering approach was used. This procedure, which is adapted from neuroimaging (Maris, 2012; Maris & Oostenveld, 2007), allows us to estimate the probability of temporally contiguous relationships being observed in our results, a fact that standard approaches to correcting for multiple comparisons fail to account for (Maris, 2012) (see also Oakes et al., 2013). In each case, the test statistic (always specified in the text) was calculated independently for each time window. Series of significant effects across contiguous time windows were identified using an alpha level of .05. A total of 1000 random datasets were then generated with the same dimensions as the original input data. Then, the same sequence of analyses was repeated, and the longest series of significant effects across contiguous time windows was identified. The results obtained from the random datasets were used to generate a histogram, and the likelihood that observed results had been obtained by chance was calculated by comparing the observed values with the randomly generated values using a standard bootstrapping procedure. Thus, a *p* value of <.01 indicates that an equivalent pattern of temporally contiguous group differences was observed in 10 or fewer of the 1000 simulated datasets created.

### Lab visit

**FIGURE 2:**
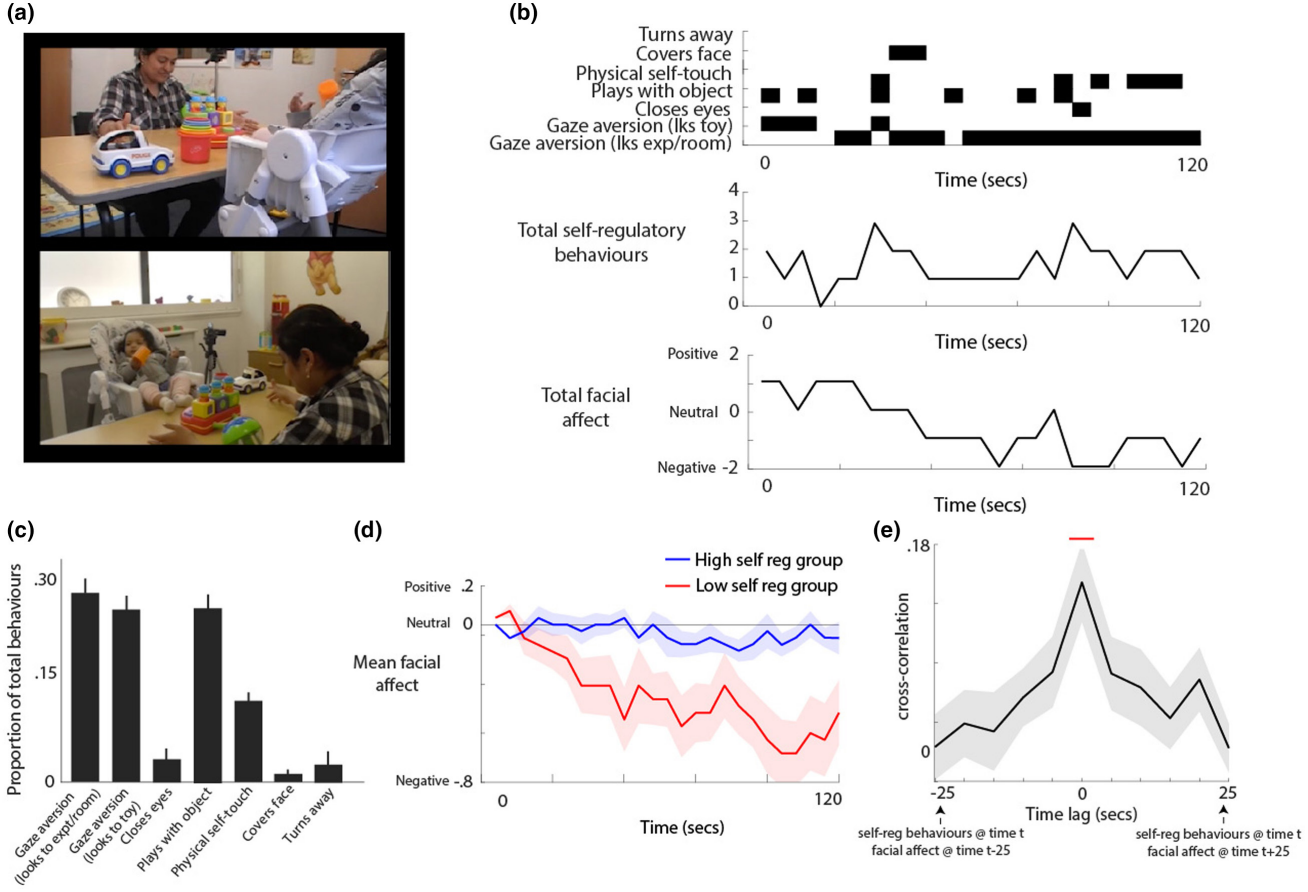
(a) Photographs showing the setup for the still‐face procedure; (b) examples of the coding of self‐regulatory behaviors; (c) bar chart showing the proportion of different self‐regulatory behaviors observed in our data. Error bars show between‐participants standard error; (d) plot showing the change in mean facial affect during the 2 min of the still‐face paradigm, subdivided by median split into the high and low self‐regulatory groups; (e) cross‐correlation showing the cross‐correlation between self‐regulatory behaviors and facial affect. Red line shows the area identified as showing a significant difference from zero in the cluster‐based permutation analysis.

The self‐regulation task was a standard version of the still‐face protocol (Weinberg & Tronick, 1996). Parent and child were seated across an 80‐cm‐wide table, and instructed to play naturally with four toys positioned on the table (see Figure 2a). After 4 min, on an instruction from the experimenter, the parent was instructed not to respond to the infant and to hold a neutral face for 2 min. On a further instruction from the experimenter, the play resumed for a further 2 min. If the infant become distressed during the still‐face period, as judged using the standard guidelines (Weinberg & Tronick, 1996), the experiment was curtailed.

Self‐regulation behaviors were coded from the NDN Physical Pilot coding scheme. Data were coded in 5‐s bins. Self‐regulatory behaviors were coded as the following categories: gaze aversion (looks to experiment/room)/gaze aversion (looks to toy)/closes eyes/plays with object/physical self‐touch/covers face/turns away (see Figure 2b). In addition, facial affect was coded on a 5‐point scale from positive (happy and engaged) to negative (fussy and irritable). Ten percent of the sample was double coded to test inter‐rater reliability. This was Cohen's kappa .71, which is acceptable.

Self‐regulation was coded as the mean number of self‐regulatory behaviors per time bin. Figure 1c shows a histogram of the results obtained. For the time‐series analyses, where it is not possible to look at group differences based on a continuous variable, data have been split using a median split. The median was 3.84. Table S1 provides a break‐down of the demographic data split by high/low self‐regulation. Associations between self‐regulation and other demographic variables are reported in the results.

In order to confirm the validity of our self‐regulation measure, we conducted two additional analyses. First, we divided our group (based on a median split as defined above) into low/high self‐regulation based on the self‐regulatory behaviors described above, and examined how the two groups differed on facial affect (Figure 2d). Second, we calculated a cross‐correlation (Figure 2e) to examine the temporal association between facial affect (coded dimensionally from negative to positive, as shown in Figure 2b) and regulatory behaviors (as also shown in Figure 2b). Areas where the cross‐correlation differed significantly from chance were identified and corrected for multiple comparisons using the same permutation‐based temporal clustering procedure as described above (see also Wass et al., 2019). A significant positive cross‐correlation was observed around lag 0, indicating a time‐specific association between increased self‐regulatory behaviors and less negative/more positive affect (Figure 2e).

Confirmatory/exploratory statement: analyses described below are relatively exploratory in nature. Analyses 1c, 2a, 2b, and 2c use methods that have not, to our knowledge, been conducted before.

## RESULTS

Our results section is in two parts. In part 1, we examine associations between lab‐assessed self‐regulation and arousal, and vocal affect and vocal intensity in home settings. In part 2, we specifically examine how responses to hyper‐ and hypo‐arousal in home settings differ contingent on lab‐assessed self‐regulation.

### Part 1—Associations between self‐regulation and arousal, and vocal affect and vocal intensity in home settings

Part 1 is in three subsections. First (part 1a), as a preliminary analysis, we present associations between self‐regulation and demographics and maternal characteristics. Then, we examine associations between self‐regulation and vocal affect and intensity (part 1b) and autonomic arousal in home settings (part 1c).

#### 1a—Demographics and maternal characteristics

For all correlations and other statistical tests, more conservative non‐parametric statistics are used throughout because not all variables were parametrically distributed. Infant self‐regulation showed a significant positive correlation with maternal occupation (*ρ* = .44, *p* = .008) but not with other demographic assessments such as maternal education (*p* = .86). No associations were observed between infant self‐regulation and maternal anxiety (*p* = .64) and depression (*p* = .09).

Poorer infant self‐regulation was associated with higher maternal vocal intensity (*ρ* = −.52, *p* = .002) but not vocal affect (*p* = .65). Better infant self‐regulation was associated with higher maternal autonomic arousal (*ρ* = .31, *p* = .04).

#### 1b—Vocal affect and intensity

**FIGURE 3:**
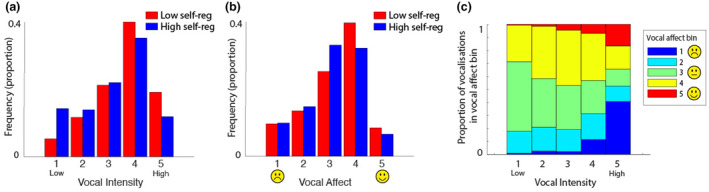
(a, b) Histograms showing the distributions of vocal intensity (a) and vocal affect (b) across all infant vocalizations recorded, subdivided by self‐regulation. (c) Stacked bar chart showing the relationship between vocal intensity (*x*‐axis) and vocal affect (color bar scale).

No associations were observed between infant self‐regulation and infant vocal intensity (*p* = .62, Figure 3a) and affect (*p* = .45, Figure 3b) in home settings. Figure 3c shows the association between vocal affect and vocal intensity. We also calculated the variability in vocal affect and intensity by concatenating all vocalizations and calculating the RMSSD. This analysis was based on a 60‐s epoch duration (which is the duration that we used for all analyses except those that specifically compare between epoch durations). Here, we observed that poorer infant self‐regulation associated with more variability in vocal affect (*ρ* = −.48, *p* = .003) and poorer infant self‐regulation was marginally associated with greater variability in vocal intensity (*ρ* = −.32, *p* = .06).

#### 1c—Autonomic arousal

**FIGURE 4:**
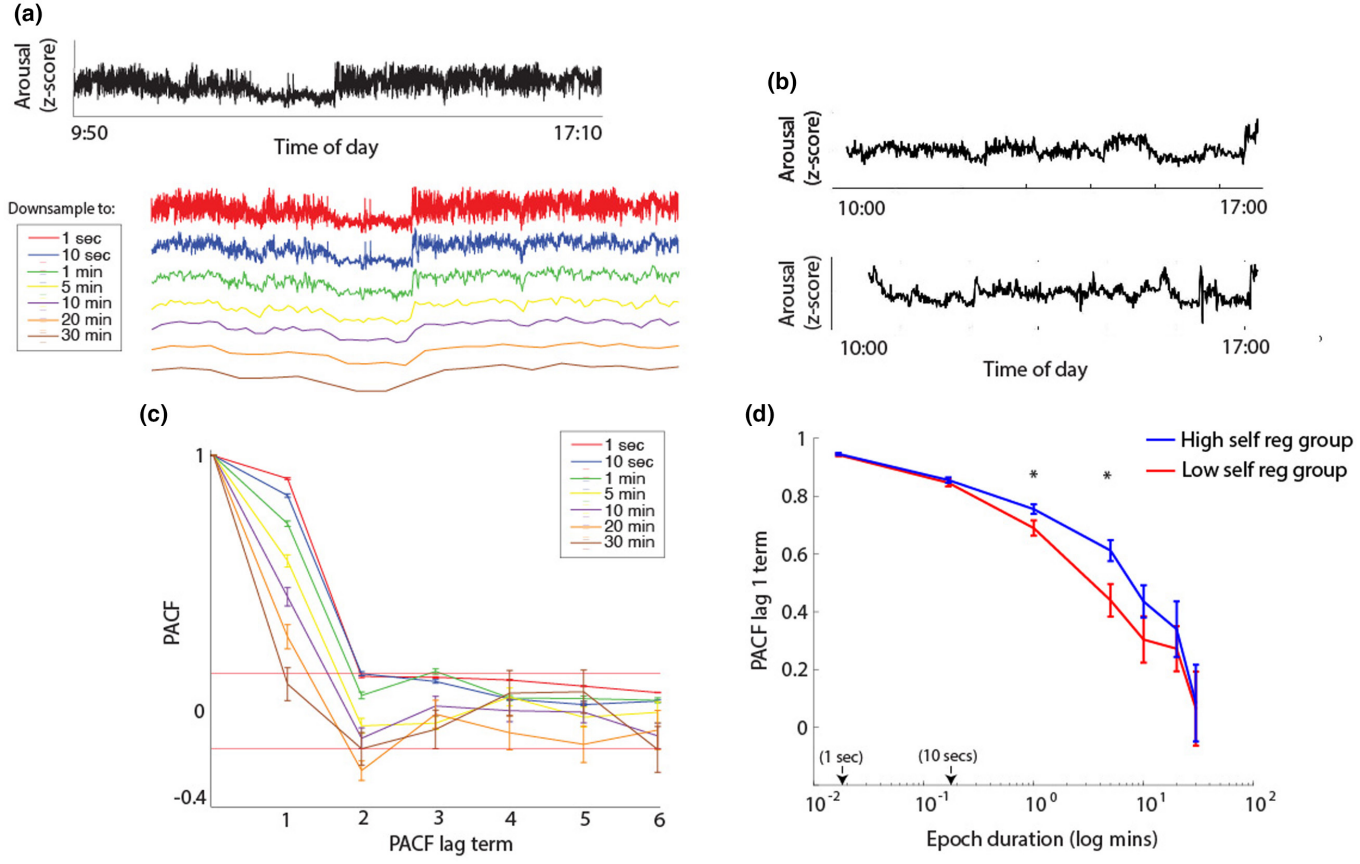
(a, b) Raw data samples to illustrate the analysis. (a) Raw autonomic arousal data from a single participant, down‐sampled using multiple different epoch durations from 1 s to 30 min. (b) Raw data samples to illustrate the partial autocorrelation function (PACF) that was calculated to index variability in the arousal data; top plot shows an example of a participant with high PACF and bottom shows an example with low PACF. (c) Line chart showing how the different lag terms of the PACF against the different epoch durations used for down‐sampling. Dotted red lines show the thresholds for significant PACF terms (positive and negative). (d) Line plot showing how the PACF lag 1 term differs between the high and low self‐regulation groups, across different epoch durations. Error bars indicate standard error of the means. *Indicates significant group difference after correcting for multiple comparisons.

No associations were observed between infant self‐regulation and infant autonomic arousal in home settings (*ρ* = .09, *p* = .58). In addition, to examine variability in autonomic arousal across multiple time scales of change in arousal, we conducted the following analysis: first, we down‐sampled the arousal data using multiple epoch durations, from 1‐s epochs through to 30‐min epochs (see Figure 4a). Next, we calculated the partial autocorrelation function (PACF). The lag 1 term of the PACF indexes the autocorrelation in the data: a high PACF term indicates greater autocorrelation (i.e., a slower rate of change in the data) (see Figure 4b). The lag 2 term indexes the autocorrelation at 2 epochs distance after the lag 1 autocorrelation has been controlled for, and so on.

Figure 4c shows the lag terms of the PACF at different epoch durations. At low epoch durations the lag 1 term of the PACF is higher, indicating that the arousal data show more autocorrelation at shorter epoch durations, as expected. (Of note, at longer epoch durations (20 and 30 min) the lag 2 term is significantly negative, pointing to low‐frequency oscillatory changes that have not to our knowledge been documented.)

Figure 4d shows just the lag 1 term of the PACF, with the different epoch durations used for the down‐sampling the data prior to calculating the autocorrelation shown on the *x*‐axis. The data have been subdivided into high and low self‐regulation groups. Permutation‐based temporal clustering analyses were used to test for group differences while correcting for multiple comparisons. Significant (*p* < .05) group differences were observed at the 1‐ and 5‐min epoch durations, indicating that the high self‐regulation group showed a slower rate of change of arousal across these time scales.

##### Part 1—Summary

Overall, these results suggest that: (i) children with higher self‐regulation show a slower rate of change of vocal intensity and affect in home settings; (ii) children with higher self‐regulation show a slower rate of change of autonomic arousal, across the 1‐ to 5‐min time scale, in home settings; and (iii) we found no evidence that children with higher lab‐assessed self‐regulation differ on mean vocal affect, intensity, and autonomic arousal in home settings.

### Part 2—Responses to hypo‐ and hyper‐arousal

In order to examine how responses to hypo‐ and hyper‐arousal differed between the low and high self‐regulation groups we conducted three analyses. First (part 2a), we used adapted Poincaré plots to examine how the stability of arousal (i.e., the likelihood of being in the same arousal bin at time *t* + 1 as at time *t*) varies as a function of arousal at time *t*. We predicted that children with better lab‐assessed self‐regulation would be more likely to show decreases in arousal following hyper‐arousal and increases in arousal following hypo‐arousal. Second (part 2b), we examined the same question in a different way, by using modeling to generate multiple simulated datasets with different parameter settings to measure which parameter setting shows the best fit for the observed data. We predicted that children with better lab‐assessed self‐regulation would show more “mean‐centering” following fluctuations above mean arousal and below mean arousal in the naturalistic home data. Third (part 2c), we examined how arousal patterns change around vocalizations, and how these relationships differed contingent on lab‐assessed self‐regulation. We predicted that, in children with better lab‐assessed self‐regulation, their autonomic arousal would be more predictive of spontaneous vocalizations, indicating that they are using vocalizations to self‐regulate.

#### 2a—Adapted Poincaré plots

First, we used adapted Poincaré plots to examine how the stability of arousal (i.e., the likelihood of being in the same arousal bin at time *t* + 1 as at time *t*) varies as a function of arousal at time *t*. We examined the likelihood of a decrease in arousal occurring at time *t* + 1 when arousal at time *t* was above the mean and compared it with the likelihood of a decrease in arousal occurring at time *t* + 1 when arousal at time *t* was below the mean. We also examined how these likelihoods differed contingent on children's lab‐assessed self‐regulation. We predicted that children with better lab‐assessed self‐regulation would be more likely to show increases in arousal following hypo‐arousal as well as decreases in arousal following hyper‐arousal.

**FIGURE 5:**
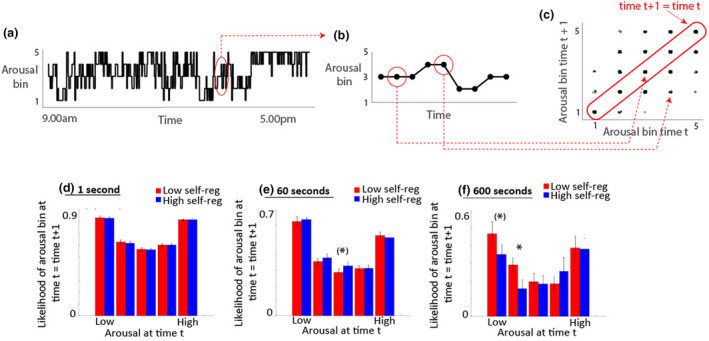
(a) Raw data sample from an individual participant, which has been down‐sampled to 1‐min epochs and binned into five equally sized bins. (b) Magnified example showing one 9‐min excerpt from the day‐long recording shown in (a). (c) Adapted Poincaré plot showing arousal at time *t* (*x*‐axis) against arousal at time *t* + 1 (*y*‐axis). (d‐f) Bar charts showing how the likelihood that arousal at time *t* = arousal at time *t* + 1 varies as a function of arousal at time *t*. Error bars show standard error of the means.The three charts show the identical analysis, but based respectively on 1 second epochs (d), 60 second epochs (e) and 600 second epochs (f). *Indicates significance of group comparisons *p < .05, (*)*p* < .1.

To test this, we first down‐sampled the continuous arousal data using variable epoch durations (1, 60, and 600 s) and binned it, participant by participant, into five equally sized bins (Figure 5a). This binning was performed participant by participant in order to correct for individual differences in mean arousal between participants. These data were then visualized using an adapted Poincaré plot with arousal at time *t* on the *x*‐axis and arousal at time *t* + 1 on the *y*‐axis. Data points on the 1:1 line (highlighted in red) are at the same arousal level at time *t* + 1 as at time *t*.

Figure 5d shows how the likelihood that arousal at time *t* = arousal at time *t* + 1 varies as a function of arousal at time *t*. All of these plots have a U‐shaped pattern, indicating that extreme high and low levels of arousal are “sticky,” as noted previously within this dataset (Wass et al., 2020). Blue and red bars show the low and high self‐regulation groups. Group differences were calculated using the non‐parametric Mann–Whitney *U* test and corrected for multiple comparisons using the Benjamini–Hochberg procedure (Benjamini & Hochberg, 1995). Results indicate that no significant differences are observed at 1‐ and 60‐s epoch durations, but that at longer epoch durations (600 s), hypo‐aroused states are less stable in the high self‐regulation group. Overall, these results suggest that, across longer time scales, low arousal levels were more likely to be followed by increases in arousal in children with better lab‐assessed self‐regulation, but no differences were found around high arousal levels.

#### 2b—Modeling

Next, we used modeling to generate multiple simulated datasets with different parameter settings to test which parameter setting shows the best fit for the observed arousal data. Using dynamical modeling (cf., Cole, Ramsook, et al., 2019; Morales et al., 2018), we generated simulated datasets with differing levels of “mean‐centering” (i.e., the tendency to return to the mean following fluctuations above or below it), using different parameters to capture “mean‐centering” following fluctuations above mean arousal (i.e., how quickly above‐average arousal tended to return back down to mean levels) and below mean arousal (i.e., how quick below‐average arousal tended to return back up to mean levels). We then assessed how each of these parameters affected the goodness of fit between the simulated data and our actual observed data, separately for each participant. And, we compared how the best‐fitting parameter settings from our simulations related to individual differences on the lab self‐regulation measure, in order to assess whether children who showed more self‐regulatory behaviors on the lab task showed greater “mean‐centering”: following fluctuations above mean arousal and below mean arousal in the naturalistic home data.

This analysis was conducted on data down‐sampled into 60‐s epochs. The simulated datasets were generated using the following equation:

$$\Delta \Theta \left(t\right)=\beta \left(\mu -\Theta \left(t\right)\right)+\mathrm{\sigma W}\left(t\right),$$

where *β* = *β*_POS_ if $\mu -\Theta \left(t\right)>0$ and *β* = *β*_NEG_ if $\mu -\Theta \left(t\right)<0$.

$\Theta \left(t\right)$ is the arousal at time *t*, *β*_POS_ and *β*_NEG_ are weighting terms, μ is the mean arousal level for that participant across the day, σ is the variance in arousal for that participant, and *W* is a random Wiener process.

Thus, simulated arousal levels at time *t* + 1 are generated by combining two terms: first, random noise (generated based on the variance in arousal observed for that participant); and second, a “mean‐centering” term generated by calculating the difference between the current arousal level and the average arousal levels observed for that child that day. When the current arousal levels are above the average for that child that day, the “mean‐centering” term will be negative, and vice versa. The strength of the “mean‐centering” term is controlled by the variable β. Two β terms were used, and varied independently: *β*_POS_ for samples where the current arousal levels are above the average for that child, and a *β*_NEG_ for samples where the current arousal levels are below the average for that child. A total of 1000 random simulated datasets were generated for each level of *β*_NEG_ from 0 to 1 (in increments of .1) and for *β*_POS_ in the same increments.

**FIGURE 6:**
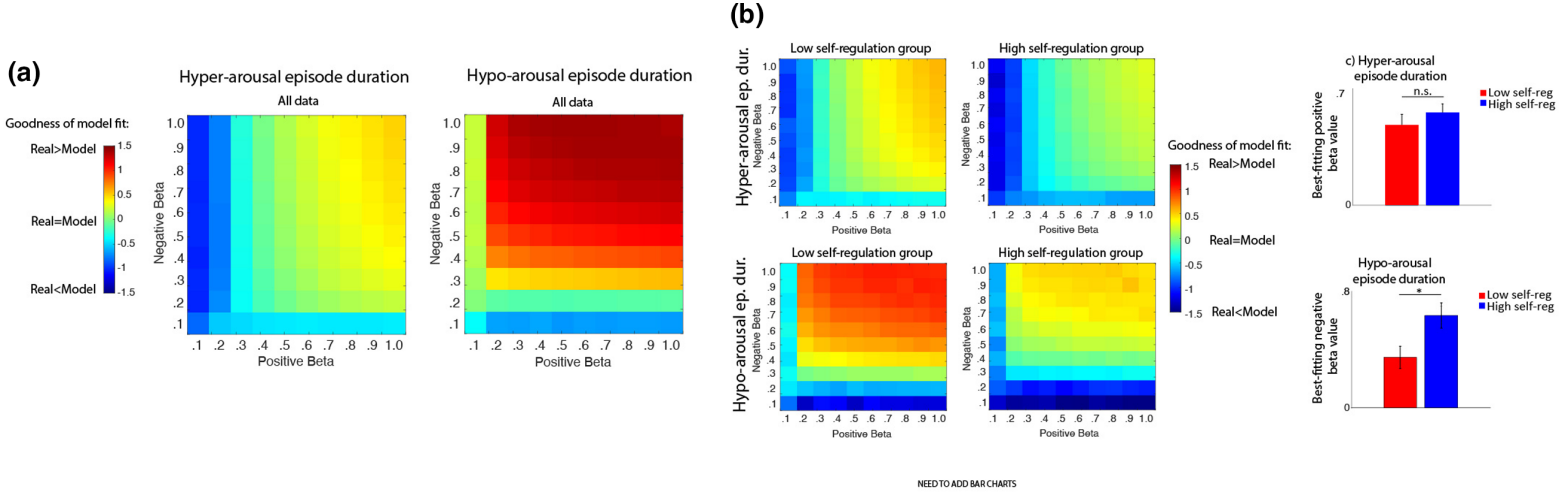
(a) Goodness of model fit for the duration of hyper‐arousal and hypo‐arousal episodes. (b) Goodness of model fit for hyper‐arousal episode duration (top) and hypo‐arousal episode duration (bottom), subdivided between the low self‐regulation group (left) and the high self‐regulation group (right). (c) Bar chart showing the best‐fitting *β*_NEG_ and *β*_POS_ values from (b), divided by self‐regulation group. Error bars indicate standard errors, and *p < .05.

Then, separately for each participant, we calculated the goodness of fit between the simulated data and the observed data, using the following procedure. The average duration of “hyper‐arousal” and “hypo‐arousal” episodes was measured by classifying each arousal epoch into five equally sized bins. This was done participant by participant in order to control for individual differences in arousal between bins. The durations of “hyper‐arousal” episodes were quantified by calculating the time intervals between arousal first exceeding the mean and returning to it, and the figure was divided by the average duration of “average arousal” episodes to control for differing levels of autocorrelation in the data. The same analysis was then repeated for each of the simulated datasets, and the average difference in “hyper‐arousal episode duration” between the observed and simulated data was calculated. The same procedure was then repeated for “hypo‐arousal episode duration.” In Figure 6a,b,d, green indicates a good fit (i.e., that the average duration of hyper‐ or hypo‐arousal episodes was similar between the observed and simulated data); red indicates that the duration of hyper‐ or hypo‐arousal episodes was longer in the real than the observed data; blue indicates the opposite. As expected, visual inspection showed that *β*_POS_ (the *x*‐axis) was more influential in determining hyper‐arousal episode duration, as shown by the fact that the colored lines on Figure 6a (left plot) are primarily vertical. *β*_NEG_ (the *y*‐axis) was more influential in determining hypo‐arousal episode duration, as shown by the fact that the lines on Figure 6a (right plot) are primarily horizontal. For hyper‐arousal episode durations (Figure 6a, left plot), the best‐fitting *β*_POS_ value was .5. For values less than .5, the model tended to underestimate hyper‐arousal episode durations (blue values); for values less than .5, it tended to overestimate them (red values). For hypo‐arousal episode durations (Figure 6a, right plot), the best‐fitting *β*_NEG_ value was .2. For values higher than this, it tended to overestimate hypo‐arousal episode durations.

Figure 6b shows the same plots split by self‐regulation group, and Figure 6c shows just the best‐fitting *β*_NEG_ values for the low and high self‐regulation groups. A Mann–Whitney U test indicated that no significant differences were observed for *β*_POS_ (“mean‐centering” following hyper‐arousal) contingent on self‐regulation *Z* = −0.99, *p* = .32. However, the best‐fitting *β*_NEG_ values (“mean‐centering” following hypo‐arousal) were significantly higher in the high self‐regulation group, *Z* = 2.6, *p* = 009.

Overall, these results suggest that “mean‐centering” (i.e., the tendency to return to mean following fluctuations above or below mean arousal) is observed more strongly in response to hypo‐arousal in the group with better lab‐assessed self‐regulation. No differences were observed around hyper‐arousal.

#### 2c—Vocalizations

Finally, we examined how predictive a child's autonomic arousal was of how likely they were to produce a cry or a speech‐like vocalization at a given moment in time. We also examined how these relationships differed contingent on lab‐assessed self‐regulation. We predicted that, in children with better lab‐assessed self‐regulation, their autonomic arousal would be more predictive of spontaneous vocalizations, indicating that they are using vocalizations to self‐regulate.

**FIGURE 7:**
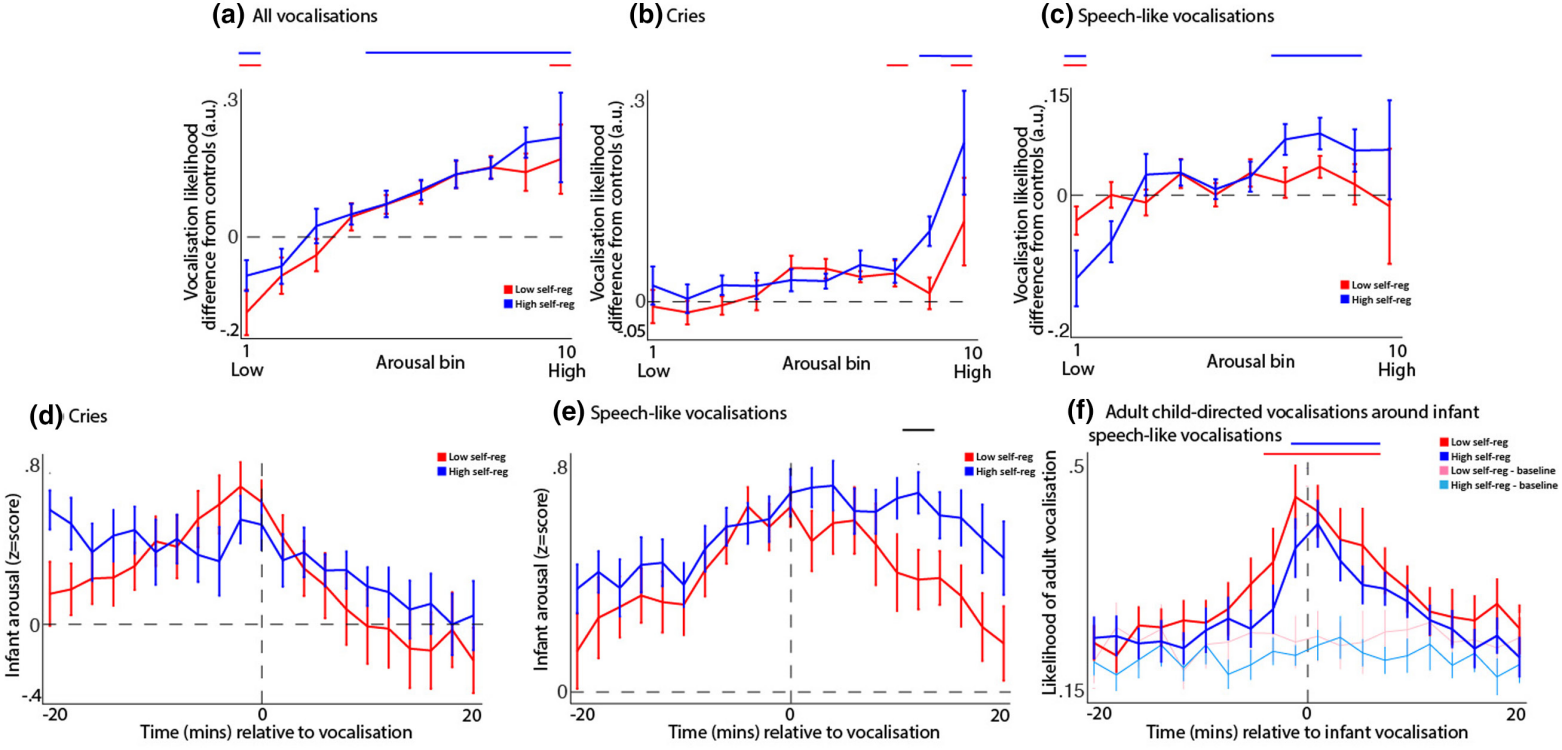
(a) Relationship between vocalization likelihood and arousal, relative to a control likelihood (calculated as described in the “Results” section). Blue and red lines above the plot show the areas of significant difference from zero in each group, after correcting for multiple comparisons. (b) Relationship between the likelihood of cries and arousal, calculated in the same way as for (a). (c) Relationship between likelihood of speech‐like vocalizations and arousal, calculated in the same way as for (a). (d) Average change in infant arousal around cries. (e) Average change in infant arousal around speech‐like vocalizations. Area highlighted with black line indicates the area of significant group difference after correcting for multiple comparisons using permutation‐based temporal clustering. (f) Change in the likelihood of an adult child‐directed vocalization around infant speech‐like vocalizations. Blue and red lines above the plot show the areas of significant difference from the baseline vocalization rates.

Vocalization likelihoods were compared with chance by comparing arousal levels at the time of observed vocalizations with arousal levels during randomly selected moments within each participant's data where no vocalization was taking place. Observed results were compared with chance using *t*‐tests, and corrected for multiple comparisons using a permutation‐based temporal clustering (see Section S1.6). Results suggested that both groups were less likely than chance to vocalize at low arousal (bin 1, Figure 7a) and more likely to vocalize at elevated arousal (bin 10, Figure 7a); but that overall, the relationship between arousal and vocalization likelihood was stronger (significant from bins 6 to 10) in the high self‐regulation group. Cries were more likely at elevated arousal in both groups (Figure 7b). Speech‐like vocalizations were less likely at low arousal in both groups (Figure 7c), but more likely at intermediate‐to‐high arousal in the high self‐regulation group only (bins 6–9).

Next, we examined how arousal levels change around vocalizations, by excerpting each participant's arousal level during the 20 min before and after each vocalization. No significant differences were identified when considering arousal levels around cries (Figure 7d). For speech‐like vocalizations, however, a sustained increase in arousal was identified during the time period after a speech‐like vocalization, in the high self‐regulation group only (Figure 7e).

Finally, we examined the likelihood that an infant's speech‐like vocalization would be preceded by a caregiver response, or elicit one in response (Figure 7f). To measure this, we examined how the observed rate of caregiver vocalizations changed during the 20 min before and after an infant speech‐like vocalization. In the low self‐regulation group, adult vocalization rates were significantly above chance during the period from 2 mins before the infant vocalization to 6 mins after; in the high self‐regulation group, they were above chance from 0 to 6 mins after (Figure 7f). Thus, infant speech‐like vocalizations were more likely to be preceded by adult child‐directed vocalizations in the low‐self‐regulation group, but not the high self‐regulation group. In both groups, infant speech‐like vocalizations were likely to be followed by adult child‐directed vocalizations.

Overall, these results suggest that the relationship between vocalization likelihood and arousal is stronger in the high self‐regulation group, and that this difference is primarily caused because speech‐like vocalizations are more concentrated around intermediate‐to‐high arousal in the high self‐regulation group. Speech‐like vocalizations are more likely to be self‐generated (i.e., not preceded by an adult child‐directed vocalization) in the high self‐regulation group.

## DISCUSSION

We used specially designed home‐wearable sensors to examine differences in how children spontaneously manifest arousal changes in home settings contingent on lab‐assessed self‐regulation. In part 1 of our analyses, we examined our data overall. We took a standard, lab‐based measure, the still‐face protocol, and video‐coded known down‐regulatory behaviors such as gaze aversion and physical self‐touch (Figure 2b,c). We split our group using a median split into one group who showed more self‐regulatory behaviors and another group who showed fewer, and we validated this approach by showing that children who used fewer self‐regulatory behaviors also showed more negative facial affect (Figure 2d). In addition, we used cross‐correlations to show that, during times when the child was using more self‐regulatory behaviors, they showed decreased negative/increased positive affect (Figure 2e).

We then examined how children with better self‐regulation in lab settings showed altered behaviors in home settings. We predicted that increased lab‐assessed self‐regulation would associate with: reduced overall arousal in naturalistic settings; reduced vocal affect and intensity in real‐world settings; a slower rate of change of vocal affect, intensity, and arousal in naturalistic settings; and faster recovery following “peak” arousal events. Overall, we found evidence that better lab‐assessed self‐regulation associated with differences in variability and the rate of change of affect and arousal in naturalistic settings (compare Somers, Luecken, et al., 2021; Somers & Luecken, 2022). Specifically, children with better lab‐assessed self‐regulation showed more variability in affect in their spontaneous vocal behaviors and a slower rate of change of autonomic arousal across multiple time scales (Figure 4d). However, we found no evidence for associations between self‐regulation in the lab and average arousal levels in real‐world settings. We also found no evidence that children with better self‐regulation show overall differences in vocal affect (e.g., reduced negative affect), or reduced vocal intensity, in home settings.

In part 2 of our analyses, we examine how infants' responses and behaviors differ contingent on hypo‐ versus hyper‐arousal. We predicted that children with better lab‐assessed self‐regulation would show both more down‐regulation from hyper‐arousal and more up‐regulation from hypo‐arousal. We also examined how the relationship between vocalization likelihood and arousal differed contingent on lab‐assessed self‐regulation. We predicted that, in children with better lab‐assessed self‐regulation, their autonomic arousal would be more predictive of vocal behaviors.

In Analysis 2a we examined how the stability of arousal (i.e., the likelihood of being in the same state at time *t* + 1 as at time *t*) varied contingent on a child's arousal level at time *t*, and their lab‐assessed self‐regulation (Figure 5). Strikingly, we found no evidence across any time scale that episodes of high arousal were more likely to be followed by decreases in arousal in the children who showed high lab‐assessed self‐regulation (Figure 5d–f). Across longer time scales, we did, however, find that low arousal levels were more likely to be followed by increases in arousal in children with better lab‐assessed self‐regulation (Figure 5d–f). In Analysis 2b, we tested the same idea using dynamical generative models and found a similar result. We produced simulated datasets with differing levels of “mean‐centering” (i.e., the tendency to return to the mean following fluctuations above or below it). We evaluated how “mean‐centering” differed between fluctuations above mean arousal and below it, and how this differed contingent on lab‐assessed self‐regulation. We found no differences contingent on self‐regulation when we examined mean‐centering following hyper‐arousal (Figure 6b,c); but we found increased mean‐centering following hypo‐arousal in children with better lab‐assessed self‐regulation (Figure 6b,c). Thus, children with different levels of behaviorally‐assessed self‐regulation in the lab showed clearer differences in up‐ rather than down‐regulation in naturalistic settings.

Any approach to studying homeostasis and allostasis in naturalistic settings faces the challenge of distinguishing active allostatic processes from the mere dissipation of arousal states (Brooks et al., 2021; Davis, 1958). Here, it is relevant to note that our cohort was defined from a lab self‐regulation measure that explicitly examines active down‐regulatory behaviors such as gaze aversion and physical self‐soothing. Perhaps most informative, however, were our analyses that examined how the likelihood of infants vocalizing varied as a function of their arousal, and how these associations differed contingent on lab‐assessed self‐regulation. Vocalizations are known to be a mechanism for eliciting caregiver engagement and arousal co‐regulation (Kopp, 1982; Wass et al., 2022). We found that all infants were more likely to produce cries at elevated arousal (Figure 7b), and that changes in autonomic arousal around cries did not significantly differ between groups (Figure 7d). However, we found that, whereas the low lab‐assessed self‐regulation group generally showed no association between arousal and the probability of producing a speech‐like vocalization, the high lab‐assessed self‐regulation group did show an association, such that they were more likely to produce speech‐like vocalizations at medium‐to‐high arousal (bins 6–9 of 10) (Figure 7c). Speech‐like vocalizations were also more likely to be self‐generated (i.e., not preceded by an adult child‐directed vocalization) in the high self‐regulation group (Figure 7f). When we examined how infant arousal changed during the time windows before and after vocalizations, we found that speech‐like vocalizations were associated with longer‐lasting increases in arousal in the high self‐regulation group (Figure 7e).

Importantly, this is not an allostatic “corrective” process, insofar as vocalizations are triggered at medium‐to‐high arousal and are followed by increases in arousal. Rather, it is evidence that, even from early infancy, children use active processes to maintain arousal at an elevated state. For example, research has suggested that information that is actively elicited by children (by vocalizing, or pointing) tends to be better retained (Begus & Bonawitz, 2020; Begus & Southgate, 2018). One possibility is that, if medium‐to‐high arousal is optimal for learning (Aston‐Jones & Cohen, 2005; Wass, 2018), then children with better lab‐assessed self‐regulation may be more likely to produce speech‐like vocalizations to elicit caregiver interactions at optimal times. Future work should also investigate further the differences between prolonged expressions of high arousal associated with positive affect and prolonged high arousal associated with negative affect (Lunkenheimer et al., 2011; Somers, Luecken, et al., 2021; Somers & Luecken, 2022).

Studying early development is essential for understanding early causative pathways in developmental psychopathology, given the associations noted between early self‐regulatory behaviors and long‐term outcomes (Eisenberg et al., 2019; Kostyrka‐Allchorne et al., 2020; Nigg, 2017). But one intrinsic limitation in studying naturalistic data in this age range is the difficulty of distinguishing co‐ from self‐regulatory processes, given the close interdependency between caregivers and infants during this period (Feldman, 2007; Kopp, 1982; Lunkenheimer et al., 2020; Somers, Curci, et al., 2021; Somers, Luecken, et al., 2021; Wass et al., 2019, 2024). For example, in the still‐face procedure that we used to characterize our sample, evidence suggests that the caregiver's behavior toward their infant influences the infant's behaviors toward the parent during the still‐face (Field, 1994; Mesman et al., 2009). This questions whether the still‐face procedure is a test of an infant's self‐regulatory capacity per se, or whether it is better thought of as measuring infant–caregiver communication. Against this, and in favor of treating the infants' self‐regulatory behaviors during the still‐face as a measure of the infant's self‐regulatory capacity, are our analyses in Figure 2d which show how the change in facial affect during the still‐face differs contingent on the degree of self‐regulatory behaviors shown by the child, and Figure 2e, which shows a time‐specific association between increased self‐regulatory behaviors and less negative/more positive affect.

The same point, that self‐ and co‐regulatory processes are hard to disentangle in this age range, also applies to our analyses of the home data, where it is impossible to be sure whether group differences in how infants' arousal changes over time (that we documented, e.g., in Figures 4d, 5 and 6) arise from differences in the infants' behavior per se, or in how the caregiver interacts with the child, moment by moment. Of note, however, when we examined behaviors such as infant cries, which are known to elicit co‐regulatory behaviors in caregivers (Wass et al., 2019, 2022), we did not observe significant differences between the low and high self‐regulation groups (Figure 7b,d). And when we examined how likely infant speech‐like vocalizations were either to follow, or to be followed by, an adult infant‐directed vocalization we did not find a significant difference contingent on child self‐regulation (Figure 7f). This suggests that the individual differences we observed between infants were not measurably contingent on observable caregiver behaviors.

One further intrinsic limitation of our approach is our use of a lab‐based measure of self‐regulation to identify individual differences in self‐regulation—which assumes, for example, that individual differences in self‐regulation are entirely trait—rather than state‐level features (see Baumeister et al., 2019). Future work using more advanced dynamical modeling techniques, including phase space analyses (Dezhina et al., 2023; Lazarus et al., 2023), could directly identify individual differences in the stability of naturally occurring arousal states; and, through that, classify self‐regulation based on naturalistic data, without reference to a separate experimental measure.

Overall, our results suggest that research into how children coordinate internal and behavioral responses in response to changing environmental demands needs to address two distinct areas of individual difference: first, what an individual's optimal state is (which may differ from individual to individual, although we did not examine this here); and second, how effective an individual is at maintaining that optimal state through allostasis (Cole et al., 2020; Kopp, 1982), and in self‐generating learning opportunities that are concentrated around times when autonomic arousal is optimal for learning (see Porges, 2007). Questionnaire measures of self‐regulation may measure individual differences in the former (i.e., differences between children's optimal states), or the latter (i.e., differences between how well children are able to maintain their optimal state)—but do not differentiate between the two (Cole, Ram, et al., 2019; Cole, Ramsook, et al., 2019).

These findings also have potential therapeutic implications. They suggest that children who perform less well on laboratory measures of self‐regulation may show either reduced interoception or a reduced ability to generate behaviors that modulate the internal state contingent on changing environmental demands (Geva et al., 2013; Porges, 2007); which, in real‐world settings, affects how children up‐regulate following hypo‐arousal more than how they down‐regulate following hyper‐arousal. Future therapeutic work should consider the former as well as the latter.

## Supporting information


Data S1.

## Data Availability

Given the sensitive nature of the data included in the manuscript (personally identifiable home audio recordings of parent–child interactions), the data on which these analyses are based are only available on personal request to the corresponding author. Sharing these data will require additional approval from our ethics board before sharing. The analyses here were not preregistered. All analytical code is available freely on request to the corresponding author.
